# STsisal: a reference-free deconvolution pipeline for spatial transcriptomics data

**DOI:** 10.3389/fgene.2025.1512435

**Published:** 2025-03-03

**Authors:** Yinghao Fu, Leqi Tian, Weiwei Zhang

**Affiliations:** ^1^ School of Mathematical Information, Shaoxing University, Zhejiang, China; ^2^ Department of Biostatistics, City University of Hong Kong, Hong Kong SAR, China; ^3^ Shenzhen Research Institute of Big Data, School of Data Science, The Chinese University of Hong Kong, Shenzhen, Guangdong, China

**Keywords:** spatial transcriptome, reference-free, deconvolution algorithm, cell type composition, hyperspectral unmixing

## Abstract

Spatial transcriptomics has emerged as an invaluable tool, helping to reveal molecular status within complex tissues. Nonetheless, these techniques have a crucial challenge: the absence of single-cell resolution, resulting in the observation of multiple cells in each spatial spot. While reference-based deconvolution methods have aimed to solve the challenge, their effectiveness is contingent upon the quality and availability of single-cell RNA (scRNA) datasets, which may not always be accessible or comprehensive. In response to these constraints, our study introduces STsisal, a reference-free deconvolution method meticulously crafted for the intricacies of spatial transcriptomics (ST) data. STsisal leverages a novel approach that integrates marker gene selection, mixing ratio decomposition, and cell type characteristic matrix analysis to discern distinct cell types with precision and efficiency within complex tissues. The main idea of our method is its adaptation of the SISAL algorithm, which expertly disentangles the ratio matrix, facilitating the identification of simplices within the ST data. STsisal offers a robust means to unveil the intricate composition of cell types in spatially resolved transcriptomic data. To verify the efficacy of STsisal, we conducted extensive simulations and applied the method to real data, comparing its performance against existing techniques. Our findings highlight the superiority of STsisal, underscoring its utility in capturing the cell composition within complex tissues.

## 1 Introduction

Spatial transcriptomics (ST) has revolutionized genomics, enabling a comprehensive exploration of gene expression within intact tissue sections. It offers profound insights into tissue architecture and its biological impact (Burgess, 2019). However, achieving single-cell resolution remains challenging due to the larger diameter of mainstream ST technologies compared to individual cells. The detected points usually represent mixtures of multiple cell types (Asp et al., 2020). Consequently, decomposing cell-type mixtures into single-cell resolution has become a critical problem in ST data analysis (Liao et al., 2021).

One natural approach to address this problem is leveraging single-cell RNA sequencing (scRNA-seq) data to assist in deconvolution. Various decomposition methods based on single-cell data have been developed, such as Tangram (Biancalani et al., 2021), Cell2location (Kleshchevnikov et al., 2022), RCTD (Cable et al., 2022), and CARD (Ma and Zhou, 2022). These methods employ diverse techniques, including deep generative models, regression algorithms, optimization techniques, non-negative matrix factorization, statistical modeling, and variational inference. By integrating scRNA-seq data with spatial information, these methods can predict cell type composition and enhance the resolution of spatial transcriptomic data.

The methods above, which utilize scRNA-seq as a reference, are commonly referred to as reference-based. Typically, reference-based deconvolution methods adopt a supervised learning framework, representing each ST point as a combination of single cells in the reference data and estimating the final ratio through approximation. However, these methods heavily rely on the availability and quality of scRNA-seq data and cell type annotations. Furthermore, systematic technical differences between single-cell and ST technologies and discrepancies in single-cell type annotations present additional challenges to accurate deconvolution (Leek et al., 2010). Consequently, there has been a growing interest in developing reference-free methods that directly deconvolve ST data without needing single-cell gene expression references. One such popular reference-free method is STdeconvolve (Miller et al., 2022), which builds on latent Dirichlet allocation (LDA). However, the performance of STdeconvolve is highly dependent on the LDA model and can be significantly affected by the quality of the ST data. For instance, sparse ST data, a limited number of spatial spots, or a lack of heterogeneity across spots can all reduce the accuracy of the method.

In this study, we propose STsisal, a novel reference-free deconvolution method designed explicitly for ST data. STsisal effectively decomposes the mixture of cell types within ST data without relying on single-cell expression reference data. The method encompasses selecting an appropriate number of cell types, the identification of loci with specific expression patterns, and the deconvolution of mixing ratios. Several methods, including the vertex component analysis (VCA) (Nascimento and Dias, 2005), Alternating Volume Maximization (AVMAX) (Ambikapathi et al., 2010), Minimum Volume Simplex Analysis (MVSA) (Li and Bioucas-Dias, 2008), and simplex identification via split augmented Lagrangian (SISAL) (Bioucas-Dias, 2009) can be employed for simplex corner identification. Extensive research has demonstrated that SISAL is more robust under various scenarios (Bioucas-Dias et al., 2012; Elkholy et al., 2020). In fact, identifying vertices of a given simplex through geometric methods can be viewed as an optimization problem with specific constraints, while SISAL uses a soft constraint or regularizer, yielding solutions that are resilient to outliers, noise, and suboptimal initialization. The cell type proportion matrix is solved using the SISAL algorithm, which aids in identifying the simplex formed by the ST data. Finally, STsisal assigns cell types based on the cell type feature matrix and known markers. Extensive simulations demonstrate that STsisal outperforms existing methods, including STdeconvolve. Moreover, in five real ST data analysis experiments, STsisal exhibits consistency with marker distributions in tissue partitions and demonstrates advantages in identifying marker genes and conducting region-based enrichment analyses.

## 2 Methods

### 2.1 STsisal overview

STsisal leverages the robust capabilities of SISAL, a linear hyperspectral unmixing technique, to unveil the intricate cellular compositions concealed within spatial transcriptomics (ST) data. Since most existing spatial technologies have yet to attain single-cell resolution, each observed spot usually contains multiple cells. To tackle this issue, our goal is to infer the cell proportion matrix for each spot using information hidden in the spatial expression matrix.

To formulate the problem mathematically, we introduce the expression matrix denoted as $Y\in R^{L\times S}$ , with L denotes the number of genes, and S represents the number of pixels. Our underlying assumption is that each pixel embodies a remarkable fusion of K distinct cell types. Thus, we can formulate the problem as Y=MH , where $M\in R^{L\times K}$ represents L genes for K cell types, and $H\in R^{K\times S}$ implies the proportions of the K cell types across S pixels. Furthermore, we impose the constraint that each column of H needs to sum up as one. This constraint ensures that each column of H represents the proportion of K cell types within a single pixel.

**FIGURE 1:**
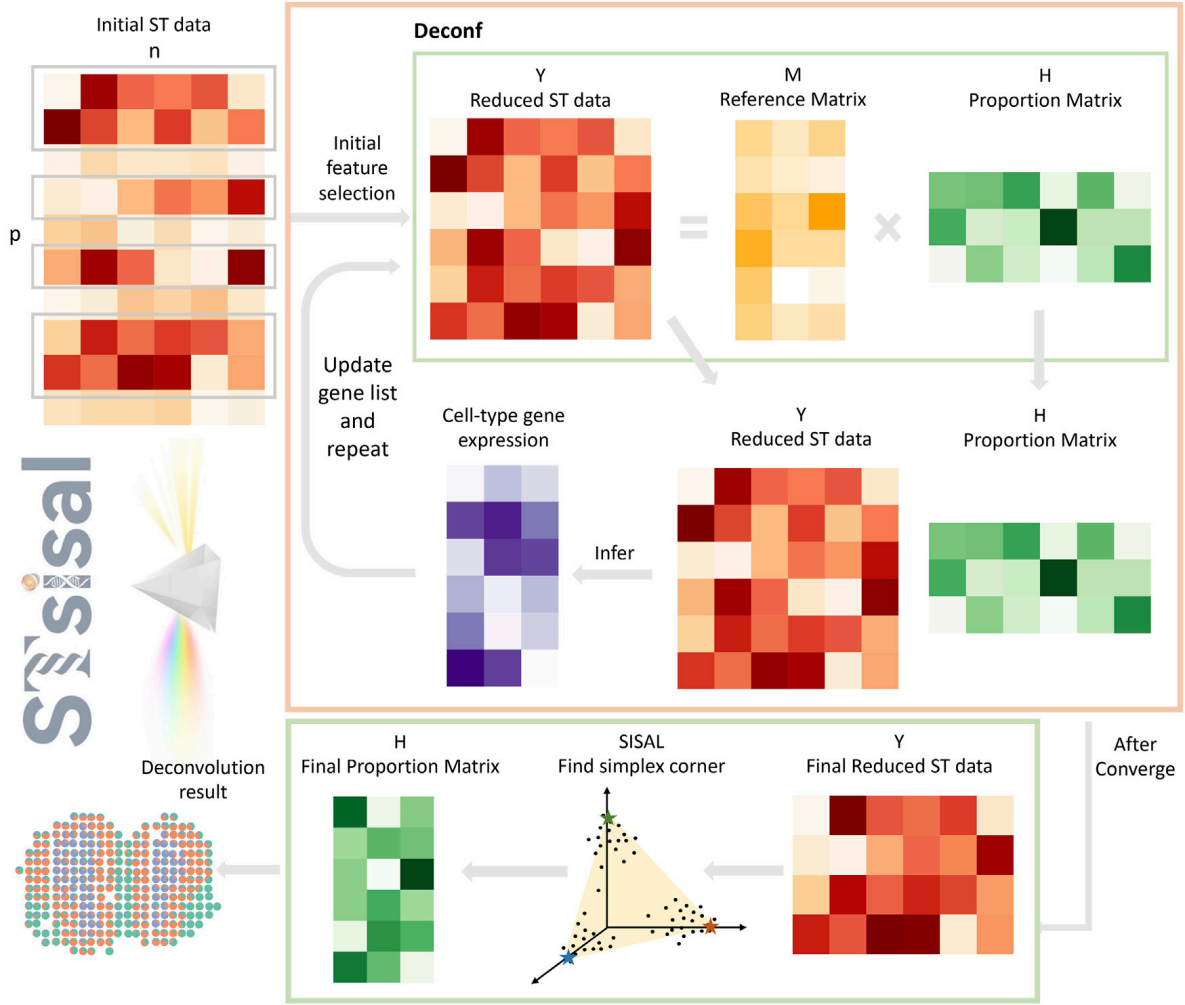
Overview of STsisal: The deconvolution process of STsisal consists of two main steps: cell type-specific gene selection (orange box) and corner identification (green box). The ST data first go through the cell type-specific gene selection step, and a feature selection procedure is applied iteratively using the deconf algorithm. After the RMSE converges, corner identification is performed using the robust algorithm SISAL.

The workflow of STsisal is depicted in Figure 1, encompassing four critical stages. Firstly, we determine the optimal number of cell types K , by utilizing a data-driven method. Secondly, once we have the estimated K , we apply a specialized cell type-specific gene selection designed for ST data to identify features specific to individual cell types. Specifically, in this step, we preprocess the original ST data into a condensed form Y through initial gene selection. The deconf algorithm is then employed to infer the reference matrix M and proportion matrix H . Based on the Y and H , we update the filtered gene list by using cross-cell type differential analysis. This iterative process continues until the root mean square error (RMSE) between the actual Y and the estimated Y=M×H converges. Thirdly, the SISAL is applied to the final reduced ST data to obtain the simplex corner and output the final proportion matrix. Lastly, in scenarios where a reference panel is accessible, we can employ a data-driven strategy to assign labels to the estimated anonymous cell types. A detailed exposition of STsial is provided in the subsequent sections.

### 2.2 Cell type number estimation

We propose a data-driven method for estimating the number of cell types present using the Akaike information criterion (AIC) for spatial transcriptomics data. This approach was also applied in epigenomic deconvolution of breast tumors (Onuchic et al., 2016), and we find it robust when analyzing spatial transcriptomics (ST) data, as evidenced by our application to the seqfish dataset in Section 3.2.5.

To identify the most suitable number of cell types K , we begin with a deconvolution algorithm to estimate cell-type-specific gene expression profiles, which we represent as $\hat{M}$ , along with the corresponding proportion matrix, denoted as $\hat{H}$ . With $\hat{M}$ and $\hat{H}$ , we proceed to evaluate the accuracy of the deconvolution process. This assessment involves calculating the sum of squared errors (SSR) between the actual observations and the reconstructed data, expressed as $SSR_{k}={\left(Y-\hat{M}\hat{H}\right)}^{2}$ . We then calculate the AIC of different K to determine the optimal one. We choose the K with the smallest AIC as the final number of cell types since the most plausible K should balance the estimated model fit and number of model complexity. For each cell type K , the AIC is calculated using the following formula:

$$AIC\left(K\right)=\left(L\times S\right)\ln\left(\frac{SSR\left(K\right)}{L\times S}\right)+2p\left(K\right)+\frac{2p\left(K\right)\left[p\left(K\right)+1\right]}{L\times S-p\left(K\right)-1}$$ (1)

where p(K)=K(L+S) is the number of parameters need to be estimated, and L×S is the number of observations in Y . The first term of the Equation 1 reflects the accuracy of the model, while the second and third terms act as penalties to discourage large increases in the number of cell types, helping to prevent overfitting. This balance between model complexity and accuracy is essential for identifying biologically meaningful cell types. However, as K increases, additional rare or subtle cell types may appear. These could represent biologically irrelevant subpopulations or artifacts introduced during the deconvolution process.

To determine the optimal K , it is recommended to calculate AIC(K) across a selected range of K values and plot K against AIC(K) . This plot often reveals a “knee” point where further increases in K provide diminishing improvements to the model fit but add unnecessary complexity. After identifying a candidate K , the inferred cell types should be evaluated for their biological relevance by checking for known markers or functional annotations. Cell types that occupy minimal proportions or lack clear biological signatures may suggest an overestimation of K and could be merged or excluded. If the selected K does not align with existing biological knowledge or the characteristics of the tissue, the range of K can be adjusted and reevaluated. This approach ensures the identification of both predominant cell types and less common groups that may hold biological significance.

### 2.3 Cell type-specific gene selection

The feature selection process plays a pivotal role in the deconvolution process and significantly impacts the accuracy of cell composition estimation. It is worth noting that marker genes often exhibit overlapping characteristics across different cell types. Consequently, employing cell type-specific markers tends to yield superior results. In contrast to methods that choose features with the highest variance, our approach emphasizes the selection of the most informative markers. The algorithm proposed in this study builds on prior research and incorporates an iterative feature selection process.

To initiate the reference-free deconvolution process from the original data matrix Y , we select an initial feature list based on the most significant coefficient of variation. This preliminary feature selection step expedites the estimation of cellular mixture proportions. To enhance the precision of the deconvolution, we utilize cross-cell type differential analysis to identify cell type-specific features using the estimated proportions. These cell type-specific features are then integrated into the successive iteration of the reference-free deconvolution process. The details of this algorithm are shown in Algorithm 1.

Algorithm 1 Feature Selection Algorithm for STsisal.
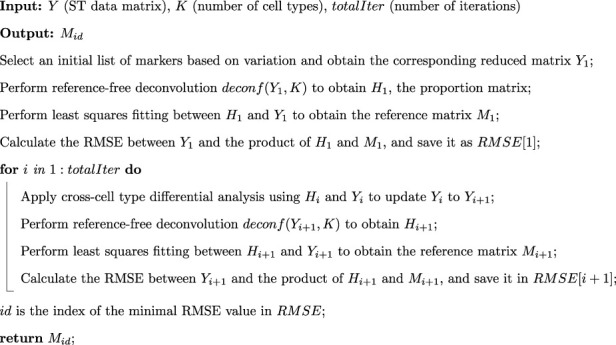

Selecting the proper reference-free deconvolution method, tailored for spatial transcriptomics data, is significant in ensuring the precision and reliability of results. To this end, we take inspiration from the algorithm used before (Repsilber et al., 2010), which offers valuable insights into the deconvolution procedure. Algorithm 2 depicts the complete process in the deconvolution step, where the iteration exit criteria were set to either iteration number < 1,000 or $\|Y-MH{\|}_{F}\leq \text{a}$ . Following this approach, we can select informative markers closer to the corners of the simplex, as discussed in the subsequent deconvolution step. Consequently, STsisal can effectively identify features that exhibit significant differences between different cell types. In the following, we retain a manageable number of genes (less than 1,000) for subsequent deconvolution analyses.

Algorithm 2 Detailed process of ***deconf*** (⋅,⋅) in the Algorithm 1.
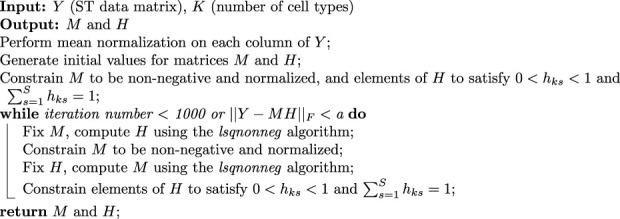

### 2.4 Deconvolution

#### 2.4.1 SISAL

The simplex identification via split augmented Lagrangian (SISAL) (Bioucas-Dias, 2009) algorithm is widely used for unsupervised hyperspectral linear unmixing. The method is formulated as Equation 2:

Y=M×H (2)

with $Y\in R^{p\times n}$ denotes the observed matrix, $M\in R^{p\times p}$ denotes the mixing matrix containing endmembers and $H=\left[h_{1},h_{2},\ldots ,h_{n}\right]\in R^{p\times n}$ represents the fraction matrix with each element in $h_{i}$ is non-negative and sum to one. With the assumption of the linear independent between each endmember $m_{i}$ , we have the set $y_{1},y_{2},\ldots ,y_{n}$ in a ( p -1)-dimensional simplex. To solve the linear unmixing problem, the equivalent optimization is solved by

$$\begin{array}{cc} M^{*}= & \arg\;\min_{M}|\det\left(M\right)|\\ \text{ s.t. } & QY⪰0,\;1_{p}^{T}QY=1_{n}^{T} \end{array}$$

with $Q=M^{-1}$ . The problem has global optima when the matrix Q is symmetric and positive-definite. However, in other cases, we can only find sub-optimal solution by solving a sequence of non-smooth convex sub-problems.

#### 2.4.2 STsisal

The deconvolution process includes simplex corner identification and proportion estimation. For convenience, we denote the spatial transcriptomics data matrix subjected to feature selection as Y in this context, and the problem can still be formulated as:

Y=M×H (3)

$$Y\in R^{L\times S},\;M\in R^{L\times K},\;H\in R^{K\times S}.$$

In Equation 3, the variables $y_{i,j}\geq 0$ describe the observed gene expression of the $i^{th}$ gene in the $j^{th}$ pixel. Similarly, $m_{i,k}\geq 0$ characterizes the gene expression of the $i^{th}$ gene in the $k^{th}$ cell type, and $h_{k,j}\geq 0$ represents the proportion of the $k^{th}$ cell type in the $j^{th}$ pixel. It is important to note that we assume that the number of distinct cell types (K) is less than the total count of pixels (S) and that the number of genes (L) is significantly greater than the number of pixels. The relationship depicts this assumption: K<S≪L . This condition ensures the following rank relationship:

$$rank\left(Y\right)=min\left(rank\left(M\right),rank\left(H\right)\right)=min\left(K,K-1\right)=K-1.$$ (4)

To maintain the non-negativity of matrix Y , row normalization is applied and we denote the normalized matrix as $\tilde{Y}$ with ${\tilde{y}}_{ij}=y_{ij}/\sum_{s=1}^{S}y_{is},\text{for }i=1,2,.,L\text{ and }j=1,2,\ldots ,S$ . This normalization ensures the sum of any row in the matrix $\tilde{Y}$ equals one (see Equation 4 for rank constraints). Then, we utilize the SISAL (Simplex Identification via Split Augmented Lagrangian) algorithm on matrix $\tilde{Y}$ to identify the corners of the standard simplex.

By employing SISAL, we can derive the pseudo proportion matrix $H_{p}$ . However, it is worth noting that this matrix may not necessarily adhere to the constraint of column sums equal to one. To address this issue, we implement the following steps. Given that the matrix $H_{p}$ represents the corners of a simplex in a space with dimensions K×S , our primary goal is to determine coefficients $\alpha_{1},\ldots ,\alpha_{K}$ that provide the best possible fit to the following equation:

$$\left[\begin{array}{c} \alpha_{1}\\ \vdots \\ \alpha_{K} \end{array}\right]\times H_{p}=c$$ (5)

where c=(1,1,…,1,1) denotes a vector of ones and matrix $H_{p}$ is denoted as:

$$H_{p}=\left[\begin{array}{ccc} h_{1,1}^{p} & \cdots & h_{1,S}^{p}\\ \vdots & \ddots & \vdots \\ h_{K,1}^{p} & \cdots & h_{K,S}^{p} \end{array}\right]$$ (6)

Subsequently, we can calculate the proportion matrix H as:

$$H=\left[\begin{array}{ccc} \alpha_{1}h_{1,1}^{p} & \cdots & \alpha_{1}h_{1,S}^{p}\\ \vdots & \ddots & \vdots \\ \alpha_{K}h_{K,1}^{p} & \cdots & \alpha_{K}h_{K,S}^{p} \end{array}\right]$$ (7)

The matrix M corresponding to matrix H then can be calculated using a rapid combinatorial non-negative least squares method. It is crucial to emphasize that the Euclidean distance between every gene and each corner is computed within the projected space following corner identification. From each corner, we choose the top G genes that are closest in proximity (see Equations 5–7 for the mathematical formulation).

### 2.5 Cell-type label assignment using data-driven approach

In this work, we introduce an advanced data-driven framework to facilitate the annotation of cell-type labels within spatial transcriptomics (ST) data. The main idea of the approach is to utilize an external reference panel comprised of cell-type annotated single-cell RNA sequencing (scRNA-seq) expression datasets. This reference allows us to compare the gene expression patterns of cell types in our deconvolution results with those in the reference dataset.

Our methodological innovation is integrating posterior probabilities derived from a Naive-Bayes classifier with Pearson correlation coefficients. These coefficients assess the congruence between the reference panel and the cell-type profiles estimated from ST data. Specifically, the Naive-Bayes classifier is employed to compute posterior probabilities by evaluating the likelihood of the observed estimated profiles under each cell type label. Concurrently, the Pearson correlation coefficient is utilized to quantify the degree of similarity between the reference and the estimated profiles.

To balance between differentiating diverse cell types and recognizing their similarity to the reference, we introduce a novel similarity scoring mechanism. This score, formulated as the mean of the correlation coefficient and posterior probability, forms the cornerstone of our cell-type prediction model. By amalgamating the correlation metrics and confidence levels derived from the classifier, our strategy significantly bolsters the precision of cell-type label assignment.

Operationally, we develop a similarity score matrix wherein each element signifies the composite similarity score pertinent to the alignment of an estimated cell type with a reference cell type. Employing an iterative selection process, we ascertain the maximal score within this matrix, facilitating allocating the column-associated predicted cell type to the row-corresponding reference cell type. Cell types exhibiting incongruence with any reference cell type or achieving a similarity score beneath a pre-established threshold are categorized as unassigned. Through this refined and systematic approach, we significantly enhance the reliability and accuracy of cell-type label assignment in spatial transcriptomics analysis.

### 2.6 Evaluation

To assess performance, we employ four metrics: RMSE (Root Mean Square Error), JSD (Jensen-Shannon Divergence), PCC (Pearson Correlation Coefficient), and MAE (Mean Absolute Error). Employing multiple metrics allows for a more comprehensive evaluation of the strengths and limitations of the assessed deconvolution methods.

The MAE is calculated as follows:

$$\text{MAE}=\frac{\sum_{i=1}^{S}\sum_{j=1}^{K}|\hat{P}ij-Pij|}{S\times K}$$ (8)

MAE is defined as the average absolute difference between the estimated proportion matrix $\left(\hat{P}\right)$ and the actual proportion matrix (P) (Equation 8). It is computed by summing the absolute differences for each spot-cell pair and dividing by the total number of spots (S) multiplied by the number of cell types (K) . By assessing the overall average bias, MAE provides a measure that complements RMSE. Whereas RMSE may be affected by individual wrongly deconvoluted spots, MAE considers the collective average discrepancy between the estimated and actual proportions.

## 3 Results and discussion

### 3.1 Simulation study

#### 3.1.1 Synthetic data generation

Our simulation study is built upon the utilization of single-cell data. Within this approach, cell selection was conducted based on a predetermined distribution extracted from a single-cell dataset. The cumulative gene expression levels of the selected cells were then amalgamated to replicate the expression profile linked with each spatial spot. The single-cell dataset employed in our study originates from the mouse nervous system (Zeisel et al., 2018), encompassing 18,263 genes and eight distinct cell types, of which six were considered: Astrocytes, Ependymal, Immune, Neurons, Oligos, and Vascular. The spatial transcriptomics data was collected from 260 spots within the mouse olfactory bulb, categorized into three different layers.

In our simulations, we modeled each anatomical region to contain a predominant cell type, reflective of typical biological structures: neurons were the main cell type in layer 1, astrocytes in layer 2, and oligodendrocytes in layer 3. To approximate the complexity of actual biological tissues, the number of co-occurring cell types in each region was determined by a uniform distribution U(2,6) . Proportions of cell types within these regions were assigned based on random draws from a Dirichlet distribution with an equal concentration parameter (α=1.0) for all included cell types.

Furthermore, we introduced a hyperparameter termed the *heterogeneity rate* to represent the proportion of spatial locations that do not conform to the dominance of a single cell type—these are the heterogeneous spots. For such spots, cell type proportions were derived from a Dirichlet distribution ( α=1.0 across all cell types) without a predefined dominant type. This rate was systematically varied from 0 to 0.6, enabling an evaluation of STsisal’s performance across a spectrum of heterogeneity scenarios.

#### 3.1.2 Simulation result

We utilized simulated ST data from the MOB dataset to compare the performance of STsisal with existing deconvolution methods such as STdeconvolve (Miller et al., 2022), RCTD (Cable et al., 2022), and CARD (Ma and Zhou, 2022). RCTD and CARD are reference-based and require a single-cell transcriptomic reference for deconvolution. In this study, the original single-cell expression data employed to generate the spatial transcriptomics (ST) data were also utilized as a reference for reference-based analytical methods. We considered three different heterogeneity rates: 0, 0.3, and 0.6. Additionally, we performed multiple simulation repetitions in different scenarios (default: 10 repetitions) to capture the impact of data variation on the robustness of the methods. To address missing or dropout values, we implement a two-step approach. Initially, missing values are filled with zeros, followed by log-normalization to ensure dataset uniformity. Subsequently, gene filtering is applied to enhance analysis robustness, retaining only those genes detected in more than 5% but less than 100% of pixels.

**FIGURE 2:**
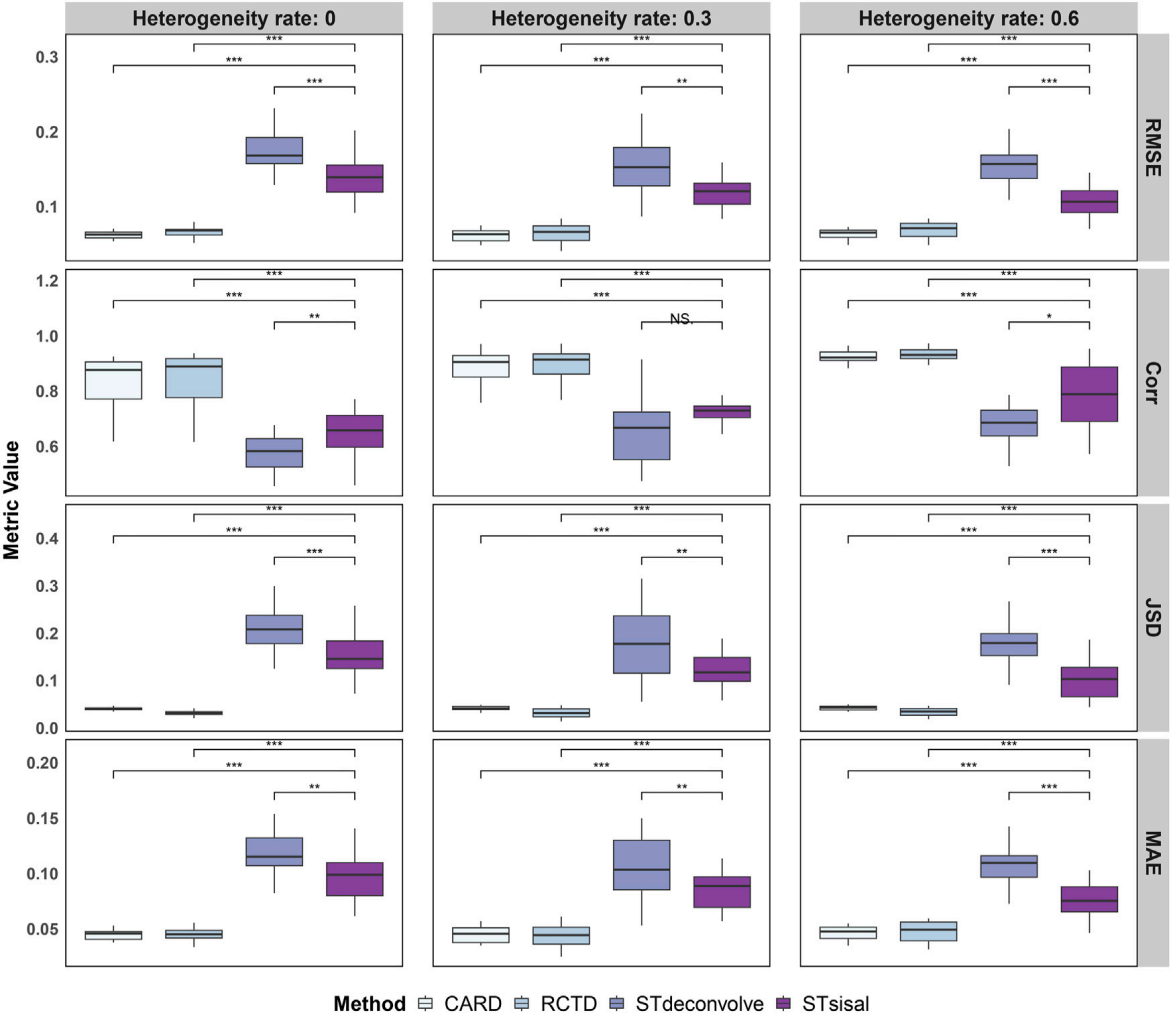
Evaluation of deconvolution performance using four different metrics, demonstrating the more robust ability of STsisal among reference-free methods and its stability compared to reference-based methods. To assess the statistical significance of the observed differences, we conducted Wilcox tests, with significance levels indicated as follows: ***: p<0.001 , **: p<0.01 , *: p<0.05 .

To evaluate the performance of these methods, we assessed four dimensions: RMSE (Root Mean Square Error), Corr (Pearson correlation coefficient), JSD (Jensen-Shannon Divergence), and MAE (Mean Absolute Error). The results are presented in Figure 2, which showcases the outcomes of the simulations. It is evident from the results that the reference-based methods outperform other approaches. This is expected as we provided an ideal single-cell reference dataset for the analysis.

Specifically, STsisal outperforms STdeconvolve in terms of various evaluation metrics. Our method exhibits lower values of RMSE, JSD, and MAE, indicating higher accuracy in predicting cell type proportions. Moreover, STsisal demonstrates a higher correlation with cell type labels, providing further evidence of its effectiveness in deconvolution tasks. Most of these results are statistically significant. While the reference-free algorithm shows slightly less robustness in the simulation experiment compared to the reference-based algorithm, it is important to note that the reference-based method relies on the availability of a perfect reference scRNA-seq profile, which is rarely attainable in real-world scenarios. This point is further supported by the real-world experiments discussed in the following sections, as reference-free methods can outperform reference-based methods when there are no matched reference data.

### 3.2 Real data applications

We used several datasets to test the performance of reference-based and reference-free methods. These datasets cover different biological systems and experimental protocols. To help readers understand the datasets used in this study, we provide a summary table (Table 1) that lists key details, including the number of genes, spatial locations or cells, data types, and the sections where each dataset is discussed.

**TABLE 1:** Summary of datasets used in real data applications.

Dataset	Protocol	#Genes	#Spatial locations/cells	Data type	Section
Mouse olfactory bulb (Replicate 12)	Spatial Transcriptomics	16,034	282	Spatial	3.2.1
GSE121891	10x Chromium	18,560	21,746	scRNA-seq	
Human PDAC	Spatial Transcriptomics	25,753	428	Spatial	3.2.2
PDAC-A	inDrop	19,736	1,926	scRNA-seq	
PDAC-B	inDrop	19,736	1,733	scRNA-seq	
Breast cancer tissue	Spatial Transcriptomics	11,920	306	Spatial	3.2.3
GSM5354515	10x Chromium	11,920	3,024	scRNA-seq	
Adult mouse brain	Spatial Transcriptomics	32,285	2,702	Spatial	3.2.4
seqFISH mouse cortex	Spatial Transcriptomics	9,684	72	Spatial	3.2.5

#### 3.2.1 STsisal identify the structures of mouse olfactory bulbs

**FIGURE 3:**
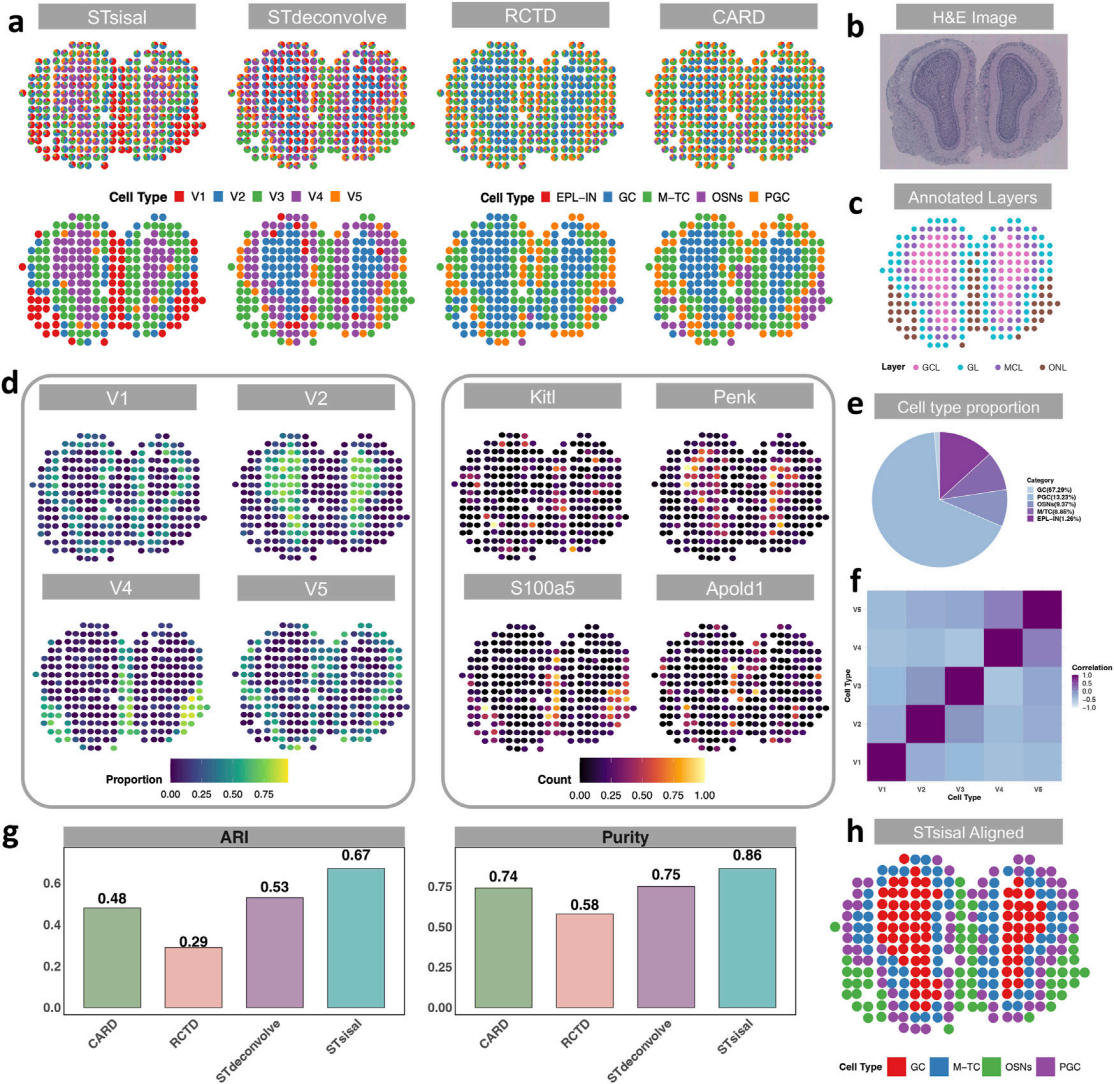
Visualization of results from actual MOB data. **(A)** Deconvolution results of actual Medial Olfactory Bulb dataset using STsisal, STdeconvolve, RCTD, and CARD. The top panel displays the cell type composition in each pixel, while the bottom panel shows the dominant cell type in each pixel. **(B, C)** Annotation of the MOB, including the H&E staining image of the MOB tissue slice (top) and the manual annotation by histologists (bottom). **(D)** Left: Proportion of cell types V1, V2, V4, and V5 inferred by STsisal displayed on each pixel. Right: Expression levels of corresponding cell-type-specific marker genes. **(E)** Cell type proportion in the scRNA reference data, where external plexiform layer interneurons (EPL-IN) only account for 1.26%. **(F)** Correlations in cell-type proportion across spatial locations between pairs of cell types inferred by STsisal. **(G)** Comparative analysis of ARI and Purity between STsisal and other methods. **(H)** Deconvolution alignment results of STsisal across five cell types.

In the previous section, we introduced the dataset of mouse olfactory bulbs (MOB) for the simulation experiment. In this section, we focused on analyzing actual spatial transcriptomics (ST) data (Ståhl et al., 2016), with the single-cell RNA sequencing (scRNA-seq) data (Tepe et al., 2018) from the same tissue serving as a reference for comparison with reference-based methods. There are five distinct cell types in the reference dataset, including granule cells (GC, n = 8,614), Olfactory sensory neurons (OSNs, n = 1,200), periglomerular cells (PGC, n = 1,693), mitral and tufted cells (M-TC, n = 1,133) external plexiform layer interneuron (EPL-IN, n = 161). Although EPL-IN has a limited amount, we still maintained it in the reference and conducted experiments with all five cell types. The MOB data consists of four distinct anatomical layers, as depicted in Figure 3C: the granule cell layer (GCL), the mitral cell layer (MCL), the glomerular layer (GL), and the nerve layer (ONL). The H&E image of the data is shown in Figure 3B. Notably, Figure 3A demonstrates that STsisal achieves the most accurate recovery of the MOB structure, whereas the other three methods tend to overlook the GL layer. While STdeconvolve can also identify this boundary, it tends to overestimate the proportions of rare cell types, such as the external plexiform layer interneurons (EPL-IN), which only account for 1.26% of the cell population as shown in Figure 3E. Moreover, the cell types inferred by STsisal exhibit clear discrimination from one another, as shown in Figure 3F. The visualization of STsisal’s aligned deconvolution results, as depicted in Figure 3H, reveals an interesting aspect of the analysis—due to the limited number of EPL-IN cells, they have been co-aligned with GC cells. Despite this, the alignment between cell type annotations and anatomical layer annotations is remarkably accurate. Analysis of the MOB dataset indicates a clear dominance of specific cell types within each olfactory layer. Utilizing the Adjusted Rand Index (ARI) and purity metrics, detailed in Figure 3G, we meticulously compared the predominant cell types inferred by STsisal against layer annotations based on H&E-stained images. This method allowed us to quantify the degree of agreement between the dominant cell types inferred by our method and the layers anatomically characterized by H&E staining.

The performance of STsisal’s estimation can be further validated by examining the cell-type composition and the expression patterns of cell-type-specific genes across different layers. The analysis operates under a crucial assumption: there exists a spatial correlation between specific cell types and their corresponding cell type-specific genes. For example, STsisal effectively classifies the boundary between the GCL and ONL layers and identifies corresponding spatially variable genes (SVGs) like Kitl, Penk, S100a5, and Apold1, whose spatial distributions closely align with the dominant cell type distribution in each layer shown in Figure 3D. Additionally, the proportion of each cell type, deconvolved by STsisal, exhibits distinct spatial colocalization patterns, facilitating clear classification of individual cell types. Although the reference-free method may not perform as well as reference-based methods in simulation experiments where a perfect reference is available, the results presented in this study clearly showcase the superior performance of STsisal in recovering the structure and accurately classifying cell types within the mouse olfactory bulbs dataset. Indeed, it is crucial to acknowledge the potential limitations of reference-based methods when the quality of the true reference data is not optimal. This recognition emphasizes the significance of reference-free methods, which offer distinct advantages in scenarios where obtaining a high-quality reference is challenging or unattainable. By circumventing the reliance on reference data, reference-free methods provide researchers with an alternative and robust approach for achieving reliable deconvolution results. STsisal, in particular, has demonstrated its effectiveness and reliability in this study, making it a valuable technique for researchers seeking accurate deconvolution results in complex datasets.

#### 3.2.2 Leveraging STsisal as a reference for selecting matched references in reference-based methods

The second dataset we examined is derived from human pancreatic ductal adenocarcinoma (PDAC) samples (Moncada et al., 2020), the most common type of pancreatic cancer known for its aggressiveness and poor prognosis. By analyzing the ST PDAC dataset, researchers aim to gain deeper insights into the spatial heterogeneity of gene expression within PDAC tumors, providing valuable information about the tumor microenvironment, cell interactions, and potential therapeutic targets.

**FIGURE 4:**
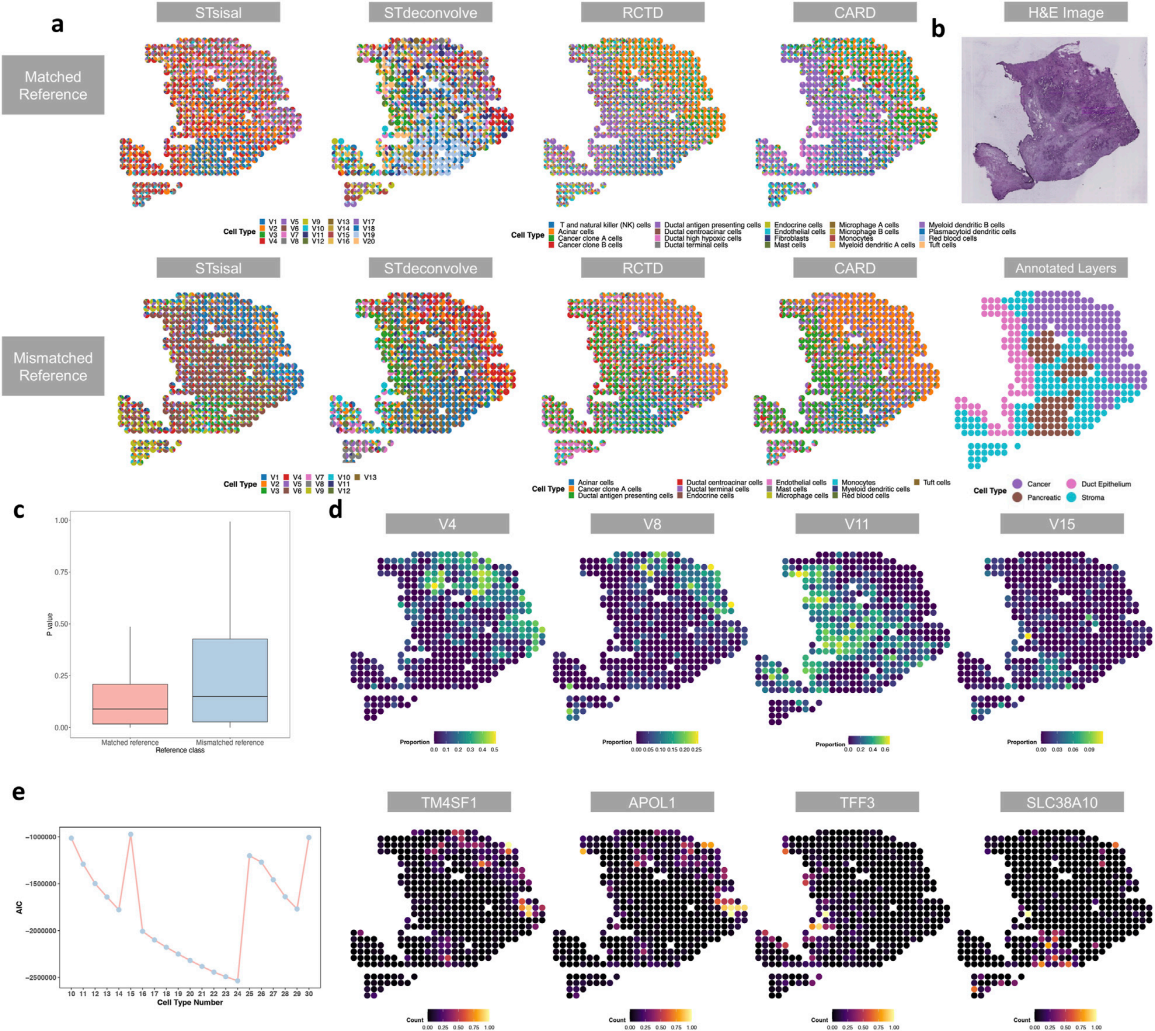
Analysis of the PDAC data. **(A)** The deconvolution results of STsisal, STdeconvolve, RCTD, and CARD using different scRNA reference data. Scatter pie plots illustrate the predicted cell-type composition by these four methods. **(B)** Annotation of the PDAC sample, including the H&E staining image of the PDAC tissue slice (top) and the manual annotation by histologists (bottom). **(C)** Comparisons of the distribution of p-values obtained from the analysis of variance (ANOVA) conducted on the markers identified by STsisal in the spatial transcriptomics (ST) data to assist in selecting appropriate reference data for reference-based methods. **(D)** The scatter pie plots display the spatial distribution of cell types, inferred by STsisal, with each spatial location showing the proportion of each cell type. Additionally, the bottom panel of the plot depicts the expression levels of corresponding marker genes specific to each cell type. **(E)** The plot depicts the Akaike Information Criterion (AIC) calculated by STsisal for varying numbers of cell types.

Histologists annotated the data into multiple tissue regions, including cancer, duct epithelium, pancreatic, and stroma, based on H&E staining images (Figure 4B). We compared four different methods: STsisal, STdeconvolve, RCTD, and CARD. For the reference-based methods, we used two reference datasets of scRNA-seq data. One dataset included 20 cell types from the same sample, while the other consisted of pancreatic single-cell data from another sample, encompassing 13 cell types. Figure 4A displays the deconvolution results of the four methods under two settings. It can be observed that STsisal accurately distinguishes cancerous regions from non-cancerous regions and separates pancreatic regions from stromal regions. In contrast, reference-based methods perform better when the reference data is matched, but their accuracy significantly decreases when the reference data is mismatched. On the other hand, reference-free methods are not affected by reference data and exhibit robustness across different numbers of cell types. When assigning varying numbers of cell types, the decomposition results of STsisal consistently align with manually annotated regions. Figure 4E shows a line graph for selecting the optimal number of cell types using STsisal. Notably, the AIC value is minimized when the number of cell types is set to 24, which is around the number of cell types in the matched reference data. This observation suggests that the model achieves an optimal fit according to the AIC criterion, highlighting the accuracy and effectiveness of STsisal in estimating the number of cell types. Figure 4C presents the p-values obtained from the analysis of variance (ANOVA) conducted on the markers identified by STsisal in the ST data. By comparing the differences in these markers across different scRNA reference datasets, we observed that the markers identified by STsisal exhibited greater statistical significance in the matched reference dataset. This finding highlights the potential of STsisal to assist reference-based methods in selecting a more suitable scRNA reference dataset. Figure 4D displays the spatial heatmaps of V4, V8, V11, and V15, along with the expression patterns of their corresponding marker genes. Based on histological images, V4 and V8 are associated with cancer, V11 is associated with duct epithelium, and V15 is associated with the pancreas.

Studies have indicated that TM4SF1 is frequently overexpressed in PDAC tumors compared to normal pancreatic tissue (Singhal et al., 2019; Zheng et al., 2015). This overexpression is associated with various malignant characteristics and poor prognosis in PDAC patients. TM4SF1 is involved in promoting tumor growth, invasion, and metastasis, contributing to the aggressiveness of PDAC. One important aspect of TM4SF1’s role in PDAC is its involvement in epithelial-mesenchymal transition (EMT). EMT is a biological process where epithelial cells lose their characteristics and acquire a mesenchymal-like phenotype, enabling them to migrate and invade surrounding tissues. TM4SF1 has been shown to induce EMT in PDAC cells, enhancing their invasiveness and metastatic potential. Moreover, TM4SF1 influences multiple signaling pathways associated with cancer progression. It can activate pathways such as PI3K/Akt, Wnt/ β -catenin, and Notch, known to regulate cell survival, proliferation, and migration. By modulating these pathways, TM4SF1 promotes PDAC cell survival, enhances their invasion into nearby tissues, and supports the formation of distant metastases. High expression of TM4SF1 is also associated with reduced sensitivity of PDAC cells to certain chemotherapy drugs, limiting the effectiveness of treatment strategies. APOL1 is involved in promoting cell proliferation, migration, invasion, and metastasis in PDAC, affecting the invasive behavior of cancer cells (Sedlakova et al., 2014). APOL1 is believed to exert its effects by modulating cell survival, apoptosis, and autophagy-related pathways. Additionally, APOL1 is associated with certain genetic variations that increase the risk of developing PDAC. It is hypothesized that these variations disrupt cellular processes and signaling pathways involved in PDAC progression.

Compared to normal duct epithelial cells, elevated levels of TFF3 are observed in PDAC tissues. This abnormal expression suggests that TFF3 may be involved in the development and progression of ductal epithelial cancer. TFF3 is involved in promoting cell proliferation, migration, invasion, and metastasis in ductal epithelial cancer. It has been shown to stimulate cell growth and activate survival pathways while inhibiting apoptosis. Additionally, TFF3 can enhance the resistance of cancer cells to chemotherapy drugs, further promoting disease progression. These effects are believed to be mediated through the activation of various signaling pathways, including MAPK and PI3K/AKT pathways (Cheng et al., 2022).

SLC38A10 is a transporter protein that regulates the transport of amino acids across cell membranes, playing a crucial role in cellular metabolism (Cheng et al., 2022). Compared to normal pancreatic tissue, elevated expression of SLC38A10 is observed in PDAC tissues. This abnormal upregulation suggests that SLC38A10 may be involved in the development and progression of PDAC. Researchers have found that increased expression of SLC38A10 promotes the uptake of essential amino acids, providing fuel for the metabolic demands of cancer cells. This enhanced amino acid transport facilitates the growth and proliferation of PDAC cells, contributing to tumor progression. Targeting SLC38A10 holds therapeutic significance for PDAC treatment. Inhibiting SLC38A10 expression or activity has been found to decrease the growth and viability of PDAC cells, indicating its potential as a novel therapeutic target for PDAC.

#### 3.2.3 Discrimination and interpretation of distinct regions in breast cancer sample using STsisal

We conducted an analysis of the 10X Visium dataset obtained from cancerous breast tissue, comprising 306 spots and 11,920 genes. Our study tested the effectiveness of various methods, namely STsisal, STdeconvolve, RCTD, and CARD. Additionally, we employed a reference-based approach using single-cell data consisting of 3,024 cells and eight cell types as the reference dataset.

**FIGURE 5:**
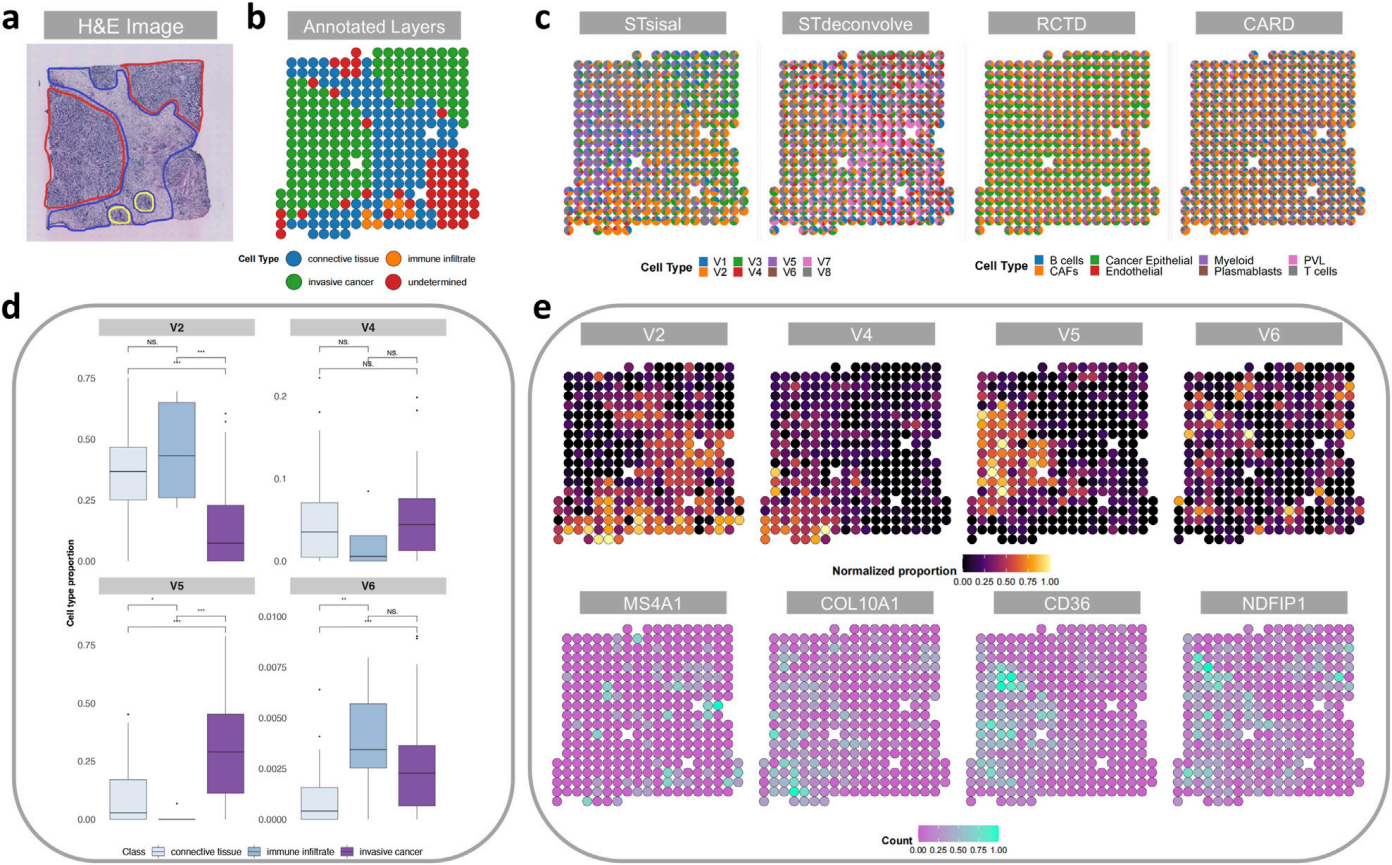
Analysis of the breast cancer data. **(A)** H&E staining image of the breast cancer tissue slice. **(B)** Manual annotation by histologists. **(C)** The spatial scatter pie plot displays inferred cell-type composition using STsisal, Stdeconvolve, RCTD, and CARD. **(D)** Comparison of cell type proportions among three distinct manual regions. **(E)** The scatter pie plots of the proportion of V2, V4, V5, V6 and the corresponding marker gene expression level.

Figure 5A showcases a histological image of the region under examination and the human-annotated classified regions (Andersson et al., 2021). Subsequently, Figure 5B presents the deconvolution results obtained from each method. Notably, our method’s deconvolution results align more closely with the human-annotated regions, particularly in distinguishing the regions associated with invasive cancer. In contrast, the results obtained from the reference-based methods were less satisfactory, likely due to limitations and biases in the quality of the single-cell reference data. Importantly, the reference-based methods failed to identify distinct regions in their deconvolution results. The robust ability of our method to precisely annotate and interpret cancer samples can greatly enhance our understanding of the underlying biology.

To further explore the differences in cell type proportions across various regions, we categorized the spots into different sets based on their respective manually annotated regions (excluding undetermined regions). Subsequently, we conducted pairwise Wilcoxon Rank Sum tests to evaluate differences in the regions where STsisal decomposed each cell type. Figure 5C depicts the boxplot of the test results, including the corresponding p-values. These results reveal significant variations in the distribution of predicted cell type proportions among different regions, thus validating the rationality of STsisal decomposition. Furthermore, Figure 5D presents the decomposed cell types and their corresponding marker genes. When comparing the heat maps of the ratio and marker gene expression patterns, we consistently observed that the matching marker gene expression patterns identified by STsisal remained in alignment, as depicted in Figure 5E.

Moreover, we performed functional enrichment analysis on the 50 marker genes associated with cell type 5, as identified by STsisal. The top terms resulting from this analysis are presented in Table 2. Upon comparing tissue images, we hypothesize that cell type V5 is related to invasive cancer. Notably, the identified important terms align with breast cancer, further substantiating the accuracy of our reverse transcription findings. Breast cancer is a complex disease characterized by dysregulated cellular processes and signaling pathways. The term “Response to growth hormone” refers to the cellular response triggered by the presence of growth hormone, which significantly impacts tumor growth and proliferation in breast cancer (Subramani et al., 2017). Gaining a comprehensive understanding of the regulation of this pathway holds promising prospects for targeted interventions. Moreover, studies have shown that breast cancer survivors are at an increased risk of cardiovascular complications following chemotherapy (Liu et al., 2023). Notably, prior to treatment, breast cancer patients often exhibit relative left ventricular hypertrophy (Maayah et al., 2020), which is closely associated with the pathway “Cardiac ventricle development.” Another important term, “Regulation of cell junction assembly,” is critical in maintaining cell-cell adhesion and tissue integrity. Dysregulation of this process may contribute to the invasive properties of breast cancer cells (Bazzoun et al., 2013). “Negative regulation of anoikis” refers to inhibiting programmed cell death due to the loss of cell-matrix interactions. This phenomenon is closely associated with increased survival and metastatic potential in breast cancer cells (Tajbakhsh et al., 2019). “Intermediate filament organization” pertains to the structural arrangement of intermediate filaments within cells, impacting cell motility, invasion, and metastasis in breast cancer (Sharma et al., 2019). Additionally, “Regulation of lipopolysaccharide-mediated signaling pathways” involves the control of immune responses triggered by lipopolysaccharides. Dysregulation of this pathway may influence inflammatory and immune evasion mechanisms in breast cancer (Wu et al., 2021), highlighting its potential as a therapeutic target.

**TABLE 2:** Top GO terms of marker genes found in V5.

GOBPID	Adjusted P-value	Term
GO:0060416	0.01188	Response to growth hormone
GO:0003231	0.01260	Cardiac ventricle development
GO:0050909	0.02640	Sensory perception of taste
GO:1901888	0.02640	Regulation of cell junction assembly
GO:2000811	0.02640	Negative regulation of anoikis
GO:1990000	0.02640	Amyloid fibril formation
GO:0001655	0.02640	Urogenital system development
GO:0045109	0.02640	Intermediate filament organization
GO:0050892	0.02640	Intestinal absorption
GO:0051497	0.02640	Negative regulation of stress fiber assembly

#### 3.2.4 STsisal demonstrates applicability to higher-resolution ST dataset

**FIGURE 6:**
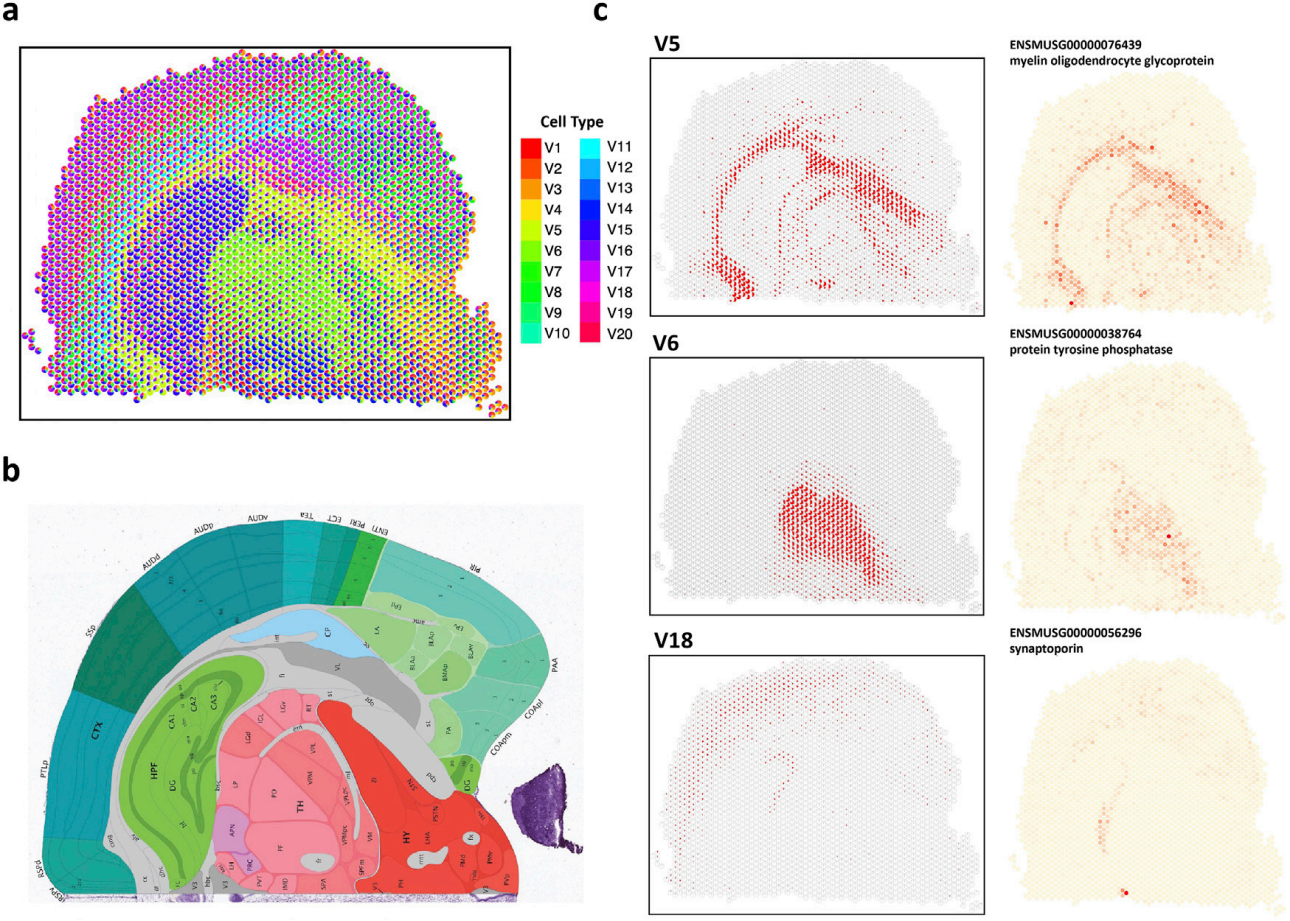
Analysis of 10X Visium mouse coronal brain data. **(A)** The spatial scatter pie plot displays inferred cell-type composition using STsisal. **(B)** Allen mouse brain atlas image with tissue type. **(C)** The scatter plot of the proportion of V5, V5, V18 and the corresponding marker gene expression level.

We next utilized Next-generation sequencing (NGS)-based data, such as 10X Visium, which provides the whole transcriptomics and achieves a resolution range from 50 $\mu m^{2}$ to 10 $\mu m^{2}$ . To explore the performance of STsisal on higher resolution data, we analyzed the 10X Visium spatial transcriptomics data of mouse coronal brain sections, which included 32,285 genes and 2,702 spots. We performed deconvolution with K=20 cell types. Figure 6A displays the results of deconvolution, while Figure 6B shows the Allen mouse brain atlas image (Lein et al., 2007). The comparison between the two figures reveals a high consistency between our deconvolution results and annotated cell types. The result indicates the successful identification of brain structures and accurate inference of the proportions of different cell types in the dataset at high resolution. Figure 6C visualizes the spatial distribution of three cell types (V5, V6, V18), which are mapped to brain fiber tracts, thalamus (TH), cerebral cortex (CTX), and pyramidal layer.

We also presented expression distribution maps of marker genes specifically identified for each cell type. Identifying marker genes further enhances our understanding of the molecular characteristics and functional roles of different cell types in the brain. Fiber tracts, also known as white matter tracts, are bundles of axons in the central nervous system that establish connections between different brain regions (Yagmurlu et al., 2016). These tracts facilitate information transmission and enable coordinated functions across various brain areas (Friederici, 2015). The relationship between fiber tracts and myelin oligodendrocyte glycoprotein (MOG) lies in MOG’s role in maintaining the integrity and stability of myelin (Buss and Schwab, 2003). Myelin is a fatty substance produced by specialized cells called oligodendrocytes. It plays a critical role in insulating and protecting axons and promoting the efficient transmission of electrical signals along the fibers (Lopez et al., 2011). Alterations in MOG expression or function can disrupt myelin stability and integrity. Abnormalities in MOG can lead to interruptions in myelin sheath formation, impairing signal transmission and altering communication between brain regions (Quarles et al., 2006).

The thalamus (TH) is a crucial structure in the brain, acting as a relay station for sensory and motor signals. It participates in various functions like perception, attention, and motor control (Ward, 2013). Protein tyrosine phosphatases (PTPs) have been found to regulate neuronal growth, differentiation, and plasticity (Zhao et al., 2022). They modulate signaling pathways associated with synaptic transmission, neuronal survival, and synaptic plasticity. PTPs have been demonstrated to regulate the development and maturation of thalamocortical connections, which are connections between the thalamus and the cerebral cortex (Bandtlow and Zimmermann, 2000). Synaptoporin is a protein primarily expressed in the brain, particularly within synapses—the junctions where neurons communicate. Synaptoporin is associated with synaptic remodeling and reorganization, especially in response to sensory input or learning experiences (Greengard et al., 1993). It participates in the dynamic changes that occur during processes like long-term potentiation (LTP) and long-term depression (LTD), which are mechanisms underlying synaptic plasticity and memory formation (Oguro-Ando et al., 2021). The connection between the cerebral cortex, particularly the pyramidal layer, and synaptoporin lies in synaptoporin’s regulatory role in synaptic transmission and plasticity in pyramidal neuron synapses.

#### 3.2.5 STsisal identifies cell type number on seqFISH data

**FIGURE 7:**
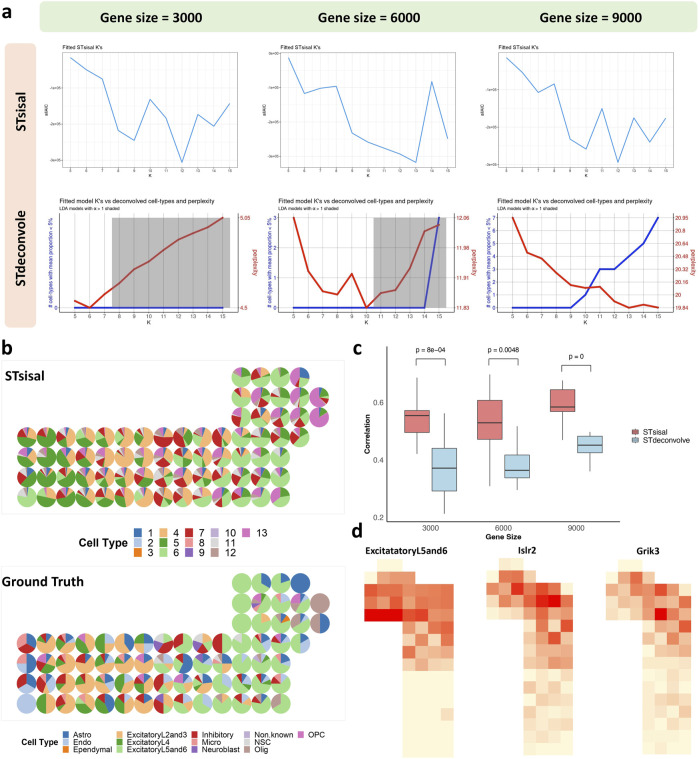
Analysis of seqFISH mouse cortex data. **(A)** The selection best cell type number K using STsisal and STdeconvolve with gene size equals 3000, 6000, and 9000. **(B)** The scatter pie plot of inferred cell-type composition using STsisal. **(C)** The scatter pie plot of ground truth cell-type composition. **(D)** The scatter plot of the ground truth (left) and STsisal-inferred (middle) proportion of ExcitatoryL5and6, along with the scatter pie plot of the corresponding marker gene expression (right).

We analyzed the seqFISH mouse cortex dataset, which consists of 524 cells corresponding to 13 cell types (Eng et al., 2019). After applying the grid-based processing described in the reference, the simulated data comprised 72 points involving 9,684 genes (Li et al., 2022). We tested two reference-free deconvolution methods, namely STsisal and STdeconvolve. We randomly sampled 3,000/6,000/9,000 gene expressions as test data for the grid-based processed data. Figure 7A displays the results of both methods in selecting the optimal number of cell types K for different gene dimensions. The first row shows the AIC values of K ranging from 5 to 15. The red line in the second row represents perplexity change (Burnham et al., 2011). Comparing these two figures, we observed that STsisal performed better in selecting K, with AIC values closer to the true value of 13. Figure 7B illustrates the transcriptional profiles of STsisal and the ground truth composition of the data in Figure 7A using a pie chart. To ensure a fair comparison, we randomly selected ten sets of different data for each gene dimension. We calculated the correlation between the results of both methods and the ground truth. Figure 7C presents the box plots describing the results of the two methods. STsisal demonstrated higher deconvolution accuracy. Furthermore, we showcased the expression distribution of marker genes identified by STsisal in their respective regions. By comparing the deconvolution results of STsisal, we determined that V6 corresponded to ExcitatoryL5 and L6. Figure 7D displays the actual distribution of ExcitatoryL5 and L6, the estimated distribution of V6 by STsisal, and the expression distribution of marker genes corresponding to V6 (Kim et al., 2015). Nptx1 is selectively expressed in subsets of excitatory neurons, including neurons in layers 5 and 6 of the cerebral cortex. Nptx1 is a protein primarily expressed in the brain, participating in various immune and inflammatory responses (Deban et al., 2009). Nptx1 is involved in synaptic plasticity in the brain, such as long-term potentiation (LTP) and long-term depression (LTD), contributing to the regulation of the balance between excitatory and inhibitory synaptic inputs (Morimoto and Nakajima, 2019).

## 4 Discussion

In this study, we introduced STsisal, a novel reference-free deconvolution framework designed to address the challenge of resolving cell-type mixtures within spatial transcriptomics (ST) spots. By leveraging the SISAL algorithm—a well-established hyperspectral unmixing method—STsisal substantially advances the resolution of ST data toward single-cell precision. Our extensive evaluations on both simulated and real-world datasets, including high-resolution platforms such as 10X Visium and seqFISH+, reveal that STsisal outperforms existing reference-free methods and achieves performance on par with reference-based approaches in scenarios where well-matched single-cell RNA sequencing (scRNA-seq) references are unavailable.

A key strength of STsisal lies in its cohesive pipeline, which integrates (1) optimal estimation of the number of cell types, (2) selection of cell-type-specific features, (3) geometric analysis for cell-type proportion estimation, and (4) cell-type label assignment. This design contrasts with methods reliant on probabilistic latent models (e.g., latent Dirichlet allocation), thereby offering greater robustness against noise and variability. The application of SISAL’s geometric principles, coupled with a targeted feature selection strategy, ensures reproducibility across diverse experimental contexts and confers resilience in detecting subtle cellular heterogeneities.

Despite its advantages, STsisal is constrained by the SISAL algorithm, specifically the requirement that the number of genes exceed the number of spots to preserve the geometric stability of the underlying simplex. While feature selection can address this limitation by reducing the dimensionality of the gene space, it entails higher computational costs and a risk of information loss. These issues become especially important for advanced ultra-high-resolution ST technologies, such as Visium HD and Stereo-seq, which generate tens of thousands of spots. Similar constraints also affect STdeconvolve, underscoring the urgent need for new reference free methods that can handle the rapidly growing scale of ST data without compromising performance.

In conclusion, STsisal provides a robust and effective solution for deconvoluting ST data without reliance on scRNA-seq references. By revealing cell-type compositions and underlying tissue architecture, it offers valuable insights into complex biological systems. While advancements to address computational demands in ultra-high-resolution ST datasets remain necessary, the framework represents a significant step forward in reference-free spatial transcriptomics analysis. Future extensions might focus on optimizing computational strategies for large-scale data, incorporating prior biological knowledge, and enabling multimodal integrative analyses. Collectively, these refinements have the potential to broaden the applicability of STsisal, further enriching our understanding of tissue organization and function across diverse experimental settings.

## Data Availability

The original contributions presented in the study are included in the article/supplementary material, further inquiries can be directed to the corresponding author.

## References

[B1] Ambikapathi A. Chan T. H. Ma W. K. Chi C. Y. (2010). A robust alternating volume maximization algorithm for endmember extraction in hyperspectral images. 2010 2nd Workshop Hyperspectral Image Signal Process. Evol. Remote Sens., 1–4. 10.1109/whispers.2010.5594862

[B2] Andersson A. Larsson L. Stenbeck L. Salmén F. Ehinger A. Wu S. Z. (2021). Spatial deconvolution of her2-positive breast cancer delineates tumor-associated cell type interactions. Nat. Commun. 12, 6012. 10.1038/s41467-021-26271-2 34650042 PMC8516894

[B3] Asp M. Bergenstråhle J. Lundeberg J. (2020). Spatially resolved transcriptomes—next generation tools for tissue exploration. BioEssays 42, 1900221. 10.1002/bies.201900221 32363691

[B4] Bandtlow C. E. Zimmermann D. R. (2000). Proteoglycans in the developing brain: new conceptual insights for old proteins. Physiol. Rev. 80, 1267–1290. 10.1152/physrev.2000.80.4.1267 11015614

[B5] Bazzoun D. Lelièvre S. Talhouk R. (2013). Polarity proteins as regulators of cell junction complexes: implications for breast cancer. Pharmacol. and Ther. 138, 418–427. 10.1016/j.pharmthera.2013.02.004 23458609 PMC3648792

[B6] Biancalani T. Scalia G. Buffoni L. Avasthi R. Lu Z. Sanger A. (2021). Deep learning and alignment of spatially resolved single-cell transcriptomes with tangram. Nat. methods 18, 1352–1362. 10.1038/s41592-021-01264-7 34711971 PMC8566243

[B7] Bioucas-Dias J. M. (2009). A variable splitting augmented Lagrangian approach to linear spectral unmixing. 2009 First Workshop Hyperspectral Image Signal Process. Evol. Remote Sens., 1–4. 10.1109/whispers.2009.5289072

[B8] Bioucas-Dias J. M. Plaza A. Dobigeon N. Parente M. Du Q. Gader P. (2012). Hyperspectral unmixing overview: geometrical, statistical, and sparse regression-based approaches. IEEE J. Sel. Top. Appl. Earth Observations Remote Sens. 5, 354–379. 10.1109/jstars.2012.2194696

[B9] Burgess D. J. (2019). Spatial transcriptomics coming of age. Nat. Rev. Genet. 20, 317. 10.1038/s41576-019-0129-z 30980030

[B10] Burnham K. P. Anderson D. R. Huyvaert K. P. (2011). Aic model selection and multimodel inference in behavioral ecology: some background, observations, and comparisons. Behav. Ecol. Sociobiol. 65, 23–35. 10.1007/s00265-010-1029-6

[B11] Buss A. Schwab M. E. (2003). Sequential loss of myelin proteins during wallerian degeneration in the rat spinal cord. Glia 42, 424–432. 10.1002/glia.10220 12730963

[B12] Cable D. M. Murray E. Zou L. S. Goeva A. Macosko E. Z. Chen F. (2022). Robust decomposition of cell type mixtures in spatial transcriptomics. Nat. Biotechnol. 40, 517–526. 10.1038/s41587-021-00830-w 33603203 PMC8606190

[B13] Cheng F. Wang X. Chiou Y. S. He C. Guo H. Tan Y. Q. (2022). Trefoil factor 3 promotes pancreatic carcinoma progression via wnt pathway activation mediated by enhanced wnt ligand expression. Cell death and Dis. 13, 265. 10.1038/s41419-022-04700-4 PMC8948291 35332126

[B14] Deban L. Bottazzi B. Garlanda C. de la Torre Y. M. Mantovani A. (2009). Pentraxins: multifunctional proteins at the interface of innate immunity and inflammation. Biofactors 35, 138–145. 10.1002/biof.21 19449441

[B15] Elkholy M. M. Mostafa M. Ebeid H. M. Tolba M. F. (2020). Comparative analysis of unmixing algorithms using synthetic hyperspectral data. Int. Conf. Adv. Mach. Learn. Technol. Appl. (AMLTA2019) 4, 945–955. 10.1007/978-3-030-14118-9_93

[B16] Eng C. H. L. Lawson M. Zhu Q. Dries R. Koulena N. Takei Y. (2019). Transcriptome-scale super-resolved imaging in tissues by RNA seqFISH. Nature 568, 235–239. 10.1038/s41586-019-1049-y 30911168 PMC6544023

[B17] Friederici A. D. (2015). White-matter pathways for speech and language processing. Handb. Clin. neurology 129, 177–186. 10.1016/B978-0-444-62630-1.00010-X 25726269

[B18] Greengard P. Valtorta F. Czernik A. J. Benfenati F. (1993). Synaptic vesicle phosphoproteins and regulation of synaptic function. Science 259, 780–785. 10.1126/science.8430330 8430330

[B19] Kim E. J. Juavinett A. L. Kyubwa E. M. Jacobs M. W. Callaway E. M. (2015). Three types of cortical layer 5 neurons that differ in brain-wide connectivity and function. Neuron 88, 1253–1267. 10.1016/j.neuron.2015.11.002 26671462 PMC4688126

[B20] Kleshchevnikov V. Shmatko A. Dann E. Aivazidis A. King H. W. Li T. (2022). Cell2location maps fine-grained cell types in spatial transcriptomics. Nat. Biotechnol. 40, 661–671. 10.1038/s41587-021-01139-4 35027729

[B21] Leek J. T. Scharpf R. B. Bravo H. C. Simcha D. Langmead B. Johnson W. E. (2010). Tackling the widespread and critical impact of batch effects in high-throughput data. Nat. Rev. Genet. 11, 733–739. 10.1038/nrg2825 20838408 PMC3880143

[B22] Lein E. S. Hawrylycz M. J. Ao N. Ayres M. Bensinger A. Bernard A. (2007). Genome-wide atlas of gene expression in the adult mouse brain. Nature 445, 168–176. 10.1038/nature05453 17151600

[B23] Li B. Zhang W. Guo C. Xu H. Li L. Fang M. (2022). Benchmarking spatial and single-cell transcriptomics integration methods for transcript distribution prediction and cell type deconvolution. Nat. methods 19, 662–670. 10.1038/s41592-022-01480-9 35577954

[B24] Li J. Bioucas-Dias J. M. (2008). Minimum volume simplex analysis: a fast algorithm to unmix hyperspectral data. IGARSS 2008 - 2008 IEEE Int. Geoscience Remote Sens. Symposium 3, III–250–III–253. 10.1109/IGARSS.2008.4779330

[B25] Liao J. Lu X. Shao X. Zhu L. Fan X. (2021). Uncovering an organ’s molecular architecture at single-cell resolution by spatially resolved transcriptomics. Trends Biotechnol. 39, 43–58. 10.1016/j.tibtech.2020.05.006 32505359

[B26] Liu C. Chen H. Guo S. Liu Q. Chen Z. Huang H. (2023). Anti-breast cancer-induced cardiomyopathy: mechanisms and future directions. Biomed. and Pharmacother. = Biomedecine and Pharmacother. 166, 115373. 10.1016/j.biopha.2023.115373 37647693

[B27] Lopez P. H. Ahmad A. S. Mehta N. R. Toner M. Rowland E. A. Zhang J. (2011). Myelin-associated glycoprotein protects neurons from excitotoxicity. J. Neurochem. 116, 900–908. 10.1111/j.1471-4159.2010.07069.x 21214567 PMC3059261

[B28] Ma Y. Zhou X. (2022). Spatially informed cell-type deconvolution for spatial transcriptomics. Nat. Biotechnol. 40, 1349–1359. 10.1038/s41587-022-01273-7 35501392 PMC9464662

[B29] Maayah Z. H. Takahara S. Alam A. S. Ferdaoussi M. Sutendra G. El-Kadi A. O. S. (2020). Breast cancer diagnosis is associated with relative left ventricular hypertrophy and elevated endothelin-1 signaling. BMC Cancer 20, 751. 10.1186/s12885-020-07217-1 32787791 PMC7425133

[B30] Miller B. F. Huang F. Atta L. Sahoo A. Fan J. (2022). Reference-free cell type deconvolution of multi-cellular pixel-resolution spatially resolved transcriptomics data. Nat. Commun. 13, 2339. 10.1038/s41467-022-30033-z 35487922 PMC9055051

[B31] Moncada R. Barkley D. Wagner F. Chiodin M. Devlin J. C. Baron M. (2020). Integrating microarray-based spatial transcriptomics and single-cell rna-seq reveals tissue architecture in pancreatic ductal adenocarcinomas. Nat. Biotechnol. 38, 333–342. 10.1038/s41587-019-0392-8 31932730

[B32] Morimoto K. Nakajima K. (2019). Role of the immune system in the development of the central nervous system. Front. Neurosci. 13, 916. 10.3389/fnins.2019.00916 31551681 PMC6735264

[B33] Nascimento J. Dias J. (2005). Vertex component analysis: a fast algorithm to unmix hyperspectral data. IEEE Trans. Geoscience Remote Sens. 43, 898–910. 10.1109/tgrs.2005.844293

[B34] Oguro-Ando A. Bamford R. A. Sital W. Sprengers J. J. Zuko A. Matser J. M. (2021). Cntn4, a risk gene for neuropsychiatric disorders, modulates hippocampal synaptic plasticity and behavior. Transl. Psychiatry 11, 106. 10.1038/s41398-021-01223-y 33542194 PMC7862349

[B35] Onuchic V. Hartmaier R. J. Boone D. N. Samuels M. L. Patel R. Y. White W. M. (2016). Epigenomic deconvolution of breast tumors reveals metabolic coupling between constituent cell types. Cell Rep. 17, 2075–2086. 10.1016/j.celrep.2016.10.057 27851969 PMC5115176

[B36] Quarles R. H. Macklin W. B. Morell P. (2006). Myelin formation, structure and biochemistry. Basic Neurochem. Mol. Cell. Med. aspects 7, 51–71.

[B37] Repsilber D. Kern S. Telaar A. Walzl G. Black G. F. Selbig J. (2010). Biomarker discovery in heterogeneous tissue samples-taking the in-silico deconfounding approach. BMC Bioinforma. 11, 27–15. 10.1186/1471-2105-11-27 PMC3098067 20070912

[B38] Sedlakova O. Svastova E. Takacova M. Kopacek J. Pastorek J. Pastorekova S. (2014). Carbonic anhydrase ix, a hypoxia-induced catalytic component of the ph regulating machinery in tumors. Front. physiology 4, 400. 10.3389/fphys.2013.00400 PMC3884196 24409151

[B39] Sharma P. Alsharif S. Fallatah A. Chung B. M. (2019). Intermediate filaments as effectors of cancer development and metastasis: a focus on keratins, vimentin, and nestin. Cells 8, 497. 10.3390/cells8050497 31126068 PMC6562751

[B40] Singhal M. Khatibeghdami M. Principe D. R. Mancinelli G. E. Schachtschneider K. M. Schook L. B. (2019). Tm4sf18 is aberrantly expressed in pancreatic cancer and regulates cell growth. PloS one 14, e0211711. 10.1371/journal.pone.0211711 30897168 PMC6428261

[B41] Ståhl P. L. Salmén F. Vickovic S. Lundmark A. Navarro J. F. Magnusson J. (2016). Visualization and analysis of gene expression in tissue sections by spatial transcriptomics. Science 353, 78–82. 10.1126/science.aaf2403 27365449

[B42] Subramani R. Nandy S. B. Pedroza D. A. Lakshmanaswamy R. (2017). Role of growth hormone in breast cancer. Endocrinology 158, 1543–1555. 10.1210/en.2016-1928 28379395

[B43] Tajbakhsh A. Rivandi M. Abedini S. Pasdar A. Sahebkar A. (2019). Regulators and mechanisms of anoikis in triple-negative breast cancer (tnbc): a review. Crit. Rev. oncology/hematology 140, 17–27. 10.1016/j.critrevonc.2019.05.009 31154235

[B44] Tepe B. Hill M. C. Pekarek B. Hunt P. J. Martin T. J. Martin J. F. (2018). Single-cell rna-seq of mouse olfactory bulb reveals cellular heterogeneity and activity-dependent molecular census of adult-born neurons. Cell Rep. 25, 2689–2703. 10.1016/j.celrep.2018.11.034 30517858 PMC6342206

[B45] Ward L. M. (2013). The thalamus: gateway to the mind. Wiley Interdiscip. Rev. Cognitive Sci. 4, 609–622. 10.1002/wcs.1256 26304267

[B46] Wu X. Qian S. Zhang J. Feng J. Luo K. Sun L. (2021). Lipopolysaccharide promotes metastasis via acceleration of glycolysis by the nuclear factor-*κ*b/snail/hexokinase3 signaling axis in colorectal cancer. Cancer and metabolism 9, 23–16. 10.1186/s40170-021-00260-x 33980323 PMC8117511

[B47] Yagmurlu K. Middlebrooks E. H. Tanriover N. Rhoton A. L. (2016). Fiber tracts of the dorsal language stream in the human brain. J. Neurosurg. 124, 1396–1405. 10.3171/2015.5.JNS15455 26587654

[B48] Zeisel A. Hochgerner H. Lönnerberg P. Johnsson A. Memic F. van der Zwan J. (2018). Molecular architecture of the mouse nervous system. Cell 174, 999–1014. 10.1016/j.cell.2018.06.021 30096314 PMC6086934

[B49] Zhao X. Xiong L. She L. Li L. Huang P. Liang G. (2022). The role and therapeutic implication of protein tyrosine phosphatases in alzheimer’s disease. Biomed. and Pharmacother. 151, 113188. 10.1016/j.biopha.2022.113188 35676788

[B50] Zheng B. Ohuchida K. Cui L. Zhao M. Shindo K. Fujiwara K. (2015). Tm4sf1 as a prognostic marker of pancreatic ductal adenocarcinoma is involved in migration and invasion of cancer cells. Int. J. Oncol. 47, 490–498. 10.3892/ijo.2015.3022 26035794

